# Comparison of dedicated digital specimen radiography with direct digital specimen mammography images

**DOI:** 10.1186/bcr2978

**Published:** 2011-11-04

**Authors:** JC Morel, V Milnes, A Iqbal, MJ Michell

**Affiliations:** 1 King's College Hospital NHS Trust, London, UK; 2 South East London BSP, London, UK

## Introduction

The objective was to compare the image quality obtained from a dedicated specimen modality with the image quality from a standard direct digital mammography unit.

## Methods

All wide local excision (WLE), vacuum-assisted 10G core biopsy and 14G core biopsy samples were imaged with a Hologic Dimensions mammography unit and a Bioptics Biovision digital specimen radiography system. WLE specimens were imaged without magnification on both systems. Biopsy specimens were imaged with magnification. Two readers assessed each set of images. The number of microcalcifications was recorded and visibility of each lesion was assessed on a four-point scale.

## Results

The total number of specimens was 97. Of these specimens, 67 contained microcalcification, 23 masses, four distortions and three masses with calcification. In 44/67 specimens, the Biovision system demonstrated >20 microcalcifications as opposed to only 24/67 with the Hologic system; this is shown to be significant with *P *= 0.001. In two of the specimens no calcification was demonstrated on the Hologic system, whereas the Biovision system demonstrated <5 in these cases. A significant difference was also shown in the conspicuity of the lesions between the two systems, with the lesions having greater conspicuity on the Biovision system (*P *= 0.027).

## Conclusion

Significantly more microcalcification is demonstrated by the Biovision system and conspicuity is significantly better. This provides increased confidence that a representative sample has been obtained at biopsy, and therefore increased diagnostic confidence.

